# Evaluating the use of in-store measures in retail food stores and restaurants in Brazil

**DOI:** 10.1590/S0034-8910.2015049005420

**Published:** 2015-10-19

**Authors:** Ana Clara Duran, Karen Lock, Maria do Rosario D O Latorre, Patricia Constante Jaime

**Affiliations:** I Programa de Pós-Graduação em Nutrição em Saúde Pública. Faculdade de Saúde Pública. Universidade de São Paulo. São Paulo, SP, Brasil; II Faculty of Public Health and Policy. London School of Hygiene and Tropical Medicine. University of London. London, UK; III Departamento de Epidemiologia. Faculdade de Saúde Pública. Universidade de São Paulo. São Paulo, SP, Brasil; IV Departamento de Nutrição. Faculdade de Saúde Pública. Universidade de São Paulo. São Paulo, SP, Brasil

**Keywords:** Food, Environment, Socioeconomic Factors, Restaurants, Validation Studies

## Abstract

**OBJECTIVE:**

To assess inter-rater reliability, test-retest reliability, and construct validity of retail food store, open-air food market, and restaurant observation tools adapted to the Brazilian urban context.

**METHODS:**

This study is part of a cross-sectional observation survey conducted in 13 districts across the city of Sao Paulo, Brazil in 2010-2011. Food store and restaurant observational tools were developed based on previously available tools, and then tested it. They included measures on the availability, variety, quality, pricing, and promotion of fruits and vegetables and ultra-processed foods. We used Kappa statistics and intra-class correlation coefficients to assess inter-rater and test-retest reliabilities in samples of 142 restaurants, 97 retail food stores (including open-air food markets), and of 62 restaurants and 45 retail food stores (including open-air food markets), respectively. Construct validity as the tool’s abilities to discriminate based on store types and different income contexts were assessed in the entire sample: 305 retail food stores, 8 fruits and vegetable markets, and 472 restaurants.

**RESULTS:**

Inter-rater and test-retest reliability were generally high, with most Kappa values greater than 0.70 (range 0.49-1.00). Both tools discriminated between store types and neighborhoods with different median income. Fruits and vegetables were more likely to be found in middle to higher-income neighborhoods, while soda, fruit-flavored drink mixes, cookies, and chips were cheaper and more likely to be found in lower-income neighborhoods.

**CONCLUSIONS:**

The measures were reliable and able to reveal significant differences across store types and different contexts. Although some items may require revision, results suggest that the tools may be used to reliably measure the food stores and restaurant food environment in urban settings of middle-income countries. Such studies can help .inform health promotion interventions and policies in these contexts.

## RESUMO

**OBJETIVO:**

Avaliar a confiabilidade intra- e inter-indivíduo e validade de construto de instrumentos de avaliação do microambiente alimentar em mercados, supermercados, feiras-livres e restaurantes adaptados ao contexto urbano brasileiro.

**MÉTODOS:**

Estudo transversal conduzido em 2010-2011 em 13 distritos do município de São Paulo, Brasil. Instrumentos de avaliação do microambiente alimentar, utilizados em outros países, foram adaptados e testados para o contexto brasileiro. Foram coletadas medidas de disponibilidade, variedade, qualidade, preço e promoção de frutas, hortaliças e alimentos ultraprocessados. Estatísticas Kappa e Coeficiente de Correlação Intraclasse foram utilizados para avaliar as confiabilidades inter- e intra-avaliador em amostra de 142 restaurantes e 97 mercados, supermercados e feiras livres e em 62 restaurantes e 97 mercados, supermercados e feiras livres, respectivamente. A validade de construto, definida como a capacidade dos instrumentos em discriminar diferentes contextos, foi avaliada na totalidade da amostra: 305 mercados e supermercados, oito feiras livres e 472 restaurantes.

**RESULTADOS:**

Os instrumentos foram considerados confiáveis, com coeficientes Kappa para as medidas inter- e intra-avaliadores, variando de 0,49 a 1,00. Eles também foram capazes de discriminar diferentes tipos de estabelecimentos e diferentes contextos socioeconômicos. Frutas e hortaliças foram mais encontradas em bairros de médio e alto nível socioeconômico, enquanto refrigerantes, refrescos em pó, biscoitos e salgadinhos foram mais encontrados em áreas de menor nível socioeconômico e apresentaram menores preços.

**CONCLUSÕES:**

As medidas são confiáveis e capazes de discriminar diferentes estabelecimentos e contextos. Embora alguns itens ainda requeiram revisão, os resultados sugerem que os instrumentos podem ser utilizados para avaliar o ambiente alimentar em áreas urbanas de países de renda média. Tais estudos podem informar políticas e intervenções para promoção da saúde nesses contextos.

## INTRODUCTION

A growing body of research suggests that greater availability of healthier foods are more likely to be found in higher-income neighborhoods,^2^ while unhealthy foods – such as soda, potato chips, fast food – are widely found in lower-income neighborhood and are, in turn, associated with food consumption^12,24^ and obesity.^4^ However, most of the available evidence comes from high-income countries, and it is unclear whether such associations hold in lower and middle-income countries. Recent South American studies have shown a greater concentration of grocery stores and fruit and vegetable street markets in higher income neighborhoods,^13,19^ and a greater availability of ultra-processed foods in lower-income neighborhoods.^22^

Studies exploring the food environment have used many methods to measure food access, such as the density and the location of stores, proximity to the nearest food store, as well as micro-level or consumer food environment measures, which include in-store measures of healthy and unhealthy food availability, variety, pricing, quality, promotion, and placement.^14^ Previously developed tools to measure the micro-level food environment have had their reliability tested.^6,15,1,8,27-29^ Noteworthy, most of them come from the United States.^15,18,27-29^ In-store measures in grocery stores have only more recently been studied in Latin American cities,^13,22^ though reliable in-stores measures for restaurants are yet to be reported for low and middle-income settings. Most of these instruments include measures of food availability and pricing,^15,18,27-29^ however fewer instruments addressed aspects of in-store marketing.^6,2,7,28^

To answer a range of questions about the micro-level food environment, and how they affect diet and obesity in Brazil, we have developed context specific food store and restaurant observational tools. The goal of this study was to assess inter-rater reliability, test-retest reliability, and construct validity of retail food store, open-air food market, and restaurant observation tools adapted to the Brazilian urban context.

## METHODS

As part of the Obesogenic Environment Study in Sao Paulo, Brazil (ESAO-SP), we developed food store, open-air food market, and restaurant observation tools, building on existing instruments and making use of input from a panel of experts in food environment from Australia, Europe, and the United States. We adapted several measures from the Nutrition Environment Measures Survey in Stores (NEMS-S),^15^ the Environmental Profile of a Community’s Health (EPOCH),^6^ Nutrition Environment Measures Survey in Restaurants (NEMS-R),^29^ and the in-store measurement tool developed by Ball et al.^2^ However, we have decided to emphasize fruits and vegetables and ultra-processed foods such as snacks and sugar-sweetened beverages to shorten our instruments when compared with tools previously available that included a larger variety of foods such as frozen meals and breads.^15^ Sugar-sweetened beverages and other ultra-processed foods are known as important contributors to energy intake in Brazil,^9^ and have been associated with obesity.^5^ Fruit and vegetable measures have become a major focus for intervention and policy,^7^ and surveillance data indicate that Brazilians do not meet the recommended levels of fruit and vegetable consumption.^30^ Also, we modified the fruit and vegetable section of previous instruments to include the 10 most frequently purchased fruits and vegetables in Sao Paulo Metropolitan Region, according to the Brazilian Household Budget Survey 2008/2009.^a^

The initial draft of ESAO Restaurant Observation Tool (ESAO-R) and ESAO Food Store Observation Tool (ESAO-S) were tested in two low-income and two high-income neighborhoods located in the city of Sao Paulo, Southeastern Brazil, in July 2010. The tools were modified based on the pre-testing’s results. A training protocol was developed before the reliability test.

The ESAO-R included availability and pricing of healthy (fruits and salads) and unhealthy foods (sugar-sweetened beverages and fries); facilitators and barriers for healthy eating at restaurants, such as combos; availability of nutrition information near the point of purchase or on the menu; and presence of indoor food marketing (signs, table tents, or other displays that highlight healthy options and/or energy-dense foods (cookies, sugar-sweetened beverages, fries, burgers) on the menu.^b^

The ESAO-S included aspects of food availability, variety, quality, pricing, signage, and promotion.^b^ We assessed the 10 most frequently purchased fruits and vegetables in Sao Paulo Metropolitan Region, and the three most frequently consumed ultra-processed foods by Brazilians: sugar-sweetened beverages, chocolate sandwich cookies, and corn chips.^1^

The quality and cost of fresh produce were assessed for the four most frequently purchased fruits and vegetables in Sao Paulo Metropolitan Region.^1^ We measured the cheapest variety found of a given fruit or vegetable. Fresh produce quality was rated as unacceptable if more than 75.0% of the available produce was bruised, old looking, overripe, or spotted; otherwise it was rated as acceptable. Cost was based on the posted prices per kilogram. When only price per unit was available, we weighed three random units, averaged their values, and calculated the price per kilogram. Variety was measured as the number of different types of fruits and vegetables within each kind, e.g., Green apple, Gala apple. We measured the variety for selected ultra-processed foods by the number of different brands available for purchase. The variety of soda and other sugar-sweetened beverages were measured by the number of different brands and flavors, e.g., Orange Fanta, Grape Fanta, Coke.

Promotion was measured by counting different signs or advertisements that promoted the purchase of fruits and vegetables or ultra-processed foods, such as signs with nutrition information, signs or other displays that encourage the purchase or the eating of such products, and discounts.

Data were collected between November 2010 and February 2011 in 52 census tracts located in 13 districts of the city of Sao Paulo, which were able to represent different possible combinations of neighborhood food environment (high and low density) and socioeconomic status (high- and low-income).

Raters (undergraduate and graduate students) assessed all food stores, open-air food markets, and restaurants within the sampled tracts. Their training consisted of four sections of two-hour instruction, followed by two to three-hour of practice. ESAO’s application manuals are available from the corresponding author upon request.

Ratings in restaurants were completed between 9:00 am and 11:30 am or 2:00 pm and 3:30 pm; in retail food stores between 9:00 am and 5:00 pm; and between 9:00 am and 11:00 am in open-air food markets (known as *feiras-livres* in Sao Paulo) in order to maintain consistency relative to stocking. Considering open-air food markets usually hold more than one food stall for a given fruit or vegetable, raters were trained to evaluate availability, variety, pricing, and quality of selected fruits and vegetables in the first stall they found when entering the market. After rating all items, raters left the market and entered it from the other end to repeat ratings. An average measure of ratings was considered in the analysis. ESAO-S was adapted to be used at open-air food markets in order to account for the repeated rating at these markets.

Restaurants were systematically classified into the following categories adapted from Saelens et al^29^ to the Brazilian context 1) full-service *à la carte *restaurants, 2) all-you-can-eat buffet restaurants, 3) per kilogram buffet restaurants (where foods are sold by weight), 4) chain fast food restaurants, 5) locally-owned fast food restaurants, 6) bars and establishments where alcohol was sold in large quantities, 7) bakeries, 8) coffee shops, and 9) ice cream shops.

Retail food stores and markets were systematically classified into the following categories, adapted from Glanz et al^15^ to the Brazilian context 1) convenience stores, 2) public-owned specialized fruit and vegetable markets or stores, 3) privately-owned specialized fruit and vegetable markets or stores, 4) open-air food markets, 5) locally-owned grocery stores or corner stores, 6) chain grocery stores, 7) chain supermarkets, and 8) delis.

Inter-rater reliability was assessed to show consistency among ratings by multiple coders.^16^ Two groups of trained raters independently visited a random sample of stores, which consisted of 30.0% of all available retail food stores in selected census tracts to complete the same set of assessments within two months. The ratings were carried out within an average of 42.6 days (standard deviation [SD] = 18.2 days). A random sample of 15.0% of all available stores was reassessed by the same raters within 45 days of the initial observation to evaluate test-retest reliability. The test-retest reliability is used to assess the consistency of a measure from one time to another. The time range between retest visits was from 7 to 45 days with a mean of 34.8 days (SD = 21.1 days).

Kappa statistics were used to evaluate the agreement of each one of the items on our tools based on the following scale: 0.81-1.00, almost perfect; 0.61-0.80, substantial; 0.41-0.60, moderate; 0.21-0.40, fair; 0-0.20, slightly poor; < 0, poor.^20^ For items with low frequencies, we used percent agreement for a more accurate reflection of reliability. Intraclass correlation coefficients were used to test both inter-rater and test-retest reliabilities of continuous variables, such as food price and variety. We considered excellent the ones above 0.75 and poor the ones below 0.40.^1^

Construct validity was assessed using the known-groups comparison method of discriminant validity,^17^ which tests whether concepts or measurements that are supposed to be unrelated are, in fact, unrelated.^17^ The tools’ component values were compared across groups of food establishments with known differences regarding the foods they carry. We expected the tools’ component values to differ significantly if they were valid. Then, we compared the value of the tools’ components according to store types and different income contexts. Retail food stores, large chain supermarkets and grocery stores, open-air food markets, and specialty fruit and vegetable stores were part of the “healthy” group; while locally-owned grocery stores or corner stores were part of the “unhealthy” group. For restaurants, the “healthy” group consisted of full-service restaurants and per kilogram buffet restaurants, and the “unhealthy” group consisted of fast food restaurants.

Finally, we hypothesized that more healthy foods would be found in higher-income neighborhoods. We thus compared the tools’ component values across tertiles of neighborhoods median household income. Such information was gathered from the Brazilian 2010 Census data.^c^ Census tracts were used as neighborhoods.

We used Chi-square or Fisher exact tests for dichotomous (yes/no) variables and t-test, ANOVA, Mann-Whitney, or Kruskal-Wallis for continuous variables, depending on the variable distribution (parametric or nonparametric). Data analyses were completed in 2013 using Stata 12.1. The Ethics Research Committee of the Faculdade de Saúde Pública of the Universidade de São Paulo determined that the present data collection did not constitute research with human subjects, thus being exempt from review.

## RESULTS

We identified 474 restaurants and 316 retail food stores and markets (including open-air fruit and vegetable markets) in the selected census tracts located across the city Figure shows all the assessed restaurants, retail food stores, and open-air food markets according to neighborhood income level. Store owners or managers did not allow us to assess one supermarket, two corner stores, and two large chain fast food restaurants, resulting in a final sample of 472 restaurants, 305 retail food stores, and eight open-air food markets. Of those, 142 restaurants, 95 retail food stores, and 2 open-air food markets were randomly chosen and assessed twice by different raters to calculate inter-rater reliability. Test-retest reliability was determined in a random sample of 62 restaurants, 43 retail food stores, and 2 open-air food markets.

**Figure:**
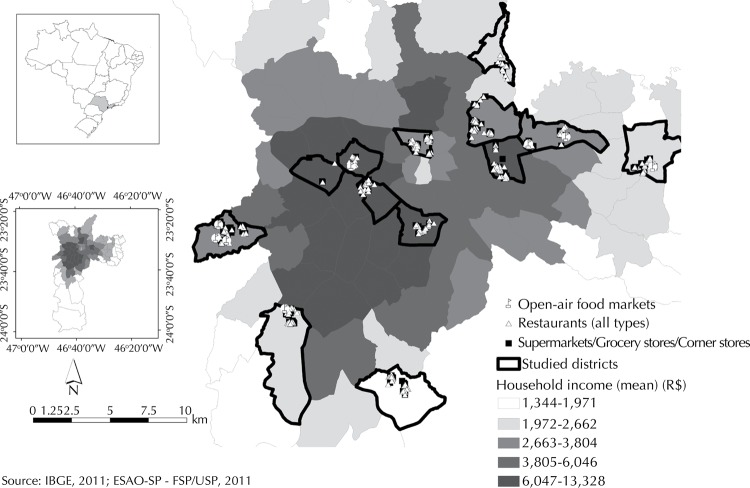
Assessed restaurants, retail food stores, and open-air food markets according to neighborhood income level. *Estudo do Ambiente Obesogênico em São Paulo* (ESAO-SP), 2011.

Mean rating time for retail food stores was 11.4 min (SD = 8.5), ranging from 1 to 50 min. For open-air food markets, mean rating time was 36.9 min (SD = 6.5), ranging from 28 to 45 min. For restaurants, mean rating time was 5.2 min (SD = 3.2), and ranged from 1 to 30 min.

Table 1 summarizes the values of inter-rater and test-retest reliability for restaurants. Kappa values for fruit and vegetable availability were generally high for both inter-rater and test-retest reliability (≥ 0.75), but lower (< 0.60) for items that indicated special requests and overeating encouragement. Some of these latter components also had low test-retest Kappa values, despite most of them being greater than 0.61.

**Table 1:** Reliability for ESAO Restaurants Observation Tool measures. Sao Paulo, Southeastern Brazil, 2011.

Item content	Inter-rater reliability (n = 142)			Test-retest reliability (n = 62)		

	n	% agreement	Kappa	n	% agreement	Kappa
Salad availability on the menu	142	82.4	0.648	62	79	0.572
Salad bar availability	142	94.4	0.776	62	93.5	0.806
Fresh fruits availability	142	91.5	0.763	62	88.7	0.669
Freshly squeezed juices availaibility	142	90.8	0.790	62	86.1	0.760
Fruits and vegetable signage/promotion	142	93.7	*	62	93.5	*
Ultra-processed foods signage	142	73.9	*	62	79.0	0.530
All-you-can-eat buffet only	142	95.8	*	62	88.7	*
Nutrition facts availability	142	99.3	0.663	62	98.4	*
Substitute fries for salad	31	78.8	*	62	62.9	0.342
Encourages smaller servings	61	75.4	0.483	29	72.6	0.455
Fruits are cheaper than or the same price as sugar-rich desserts	19	63.5	*	7	85.7	0.730
Freshly squeezed juices are cheaper than or the same price as soda	89	75.2	0.490	41	80.5	0.630
Cheaper combos	45	63.4	*	21	80.7	0.572

* Statistics could not be computed because cross tabulation had two or fewer levels.

Table 2 shows inter-rater and test-retest reliability findings for measurement tool items collected at open-air food markets and retail food stores. Kappa statistics for inter-rater reliability ranged from 0.66 to 0.95. Test-retest reliability was likewise high, with kappa statistics ranging from 0.61 to 1.00. Kappa statistics for the promotion of fruits and vegetables and ultra-processed foods were either low or could not be estimated because of limited observations.

**Table 2:** Reliability of ESAO Food Store Observation Tool measures. Sao Paulo, Southeastern Brazil, 2011.

	Inter-rater reliability (n = 97)		Test-retest reliability (n = 45)	

	% agreement	Kappa	% agreement	Kappa
Availability of any fresh fruit or vegetable	97.9	0.956	100.0	1.000
Fresh fruits and vegetable located near the entrance of the store	94.8	0.863	97.8	0.933
Availability of fresh produce				
Orange	97.9	0.951	100.0	1.000
Banana	99.0	0.956	97.8	0.945
Papaya	93.8	0.846	97.8	0.942
Apple	95.8	0.901	93.3	0.825
Tomato	94.8	0.878	97.8	0.949
Onion	99.0	0.978	97.8	0.949
Carrot	93.8	0.850	97.8	0.945
Lettuce	92.8	0.823	93.3	0.814
Quality of fresh produce				
Orange	96.1	0.809	91.1	0.788
Banana	98.8	0.943	93.3	0.838
Papaya	97.3	0.858	88.9	0.722
Apple	98.4	0.921	93.3	0.827
Tomato	94.9	0.756	88.9	0.759
Onion	96.5	0.855	91.1	0.949
Carrot	96.9	0.843	95.6	0.891
Lettuce	96.9	0.839	88.9	0.701
Availability of ultra-processed foods				
Any type of soda	95.8	0.693	100.0	1.000
Any type of sugar free soda	90.7	0.687	93.3	0.825
Sugary processed juice/nectar	89.7	0.791	86.7	0.709
Fruit-flavored drink mix	90.7	0.800	86.7	0.614
Chocolate sandwich cookies	86.6	0.583	86.7	0.715
Corn chips	92.8	0.784	84.4	0.642
Signage/Promotion				
Fruits and vegetable signage/promotion	92.8	0.549	93.3	0.536
Ultra-processed foods signage	69.1	*	88.9	*

* Statistics could not be computed because cross tabulation had two or fewer levels.

We computed infraclass correlation coefficients for quantitative components, such as food price and variety. Results regarding varieties of fruit and vegetables and ultra-processed foods were all significant, equal to or above 0.75. The price measures for restaurants and retail food stores presented excellent reliability (intraclass correlation coefficient > 0.80).

Tables 3 and 4 show results for construct validity. Our measures were able to discriminate between store types and neighborhoods of different median incomes. Full-service restaurants were more likely to have a salad bar, salads on the menu, and fresh fruits and juices, validating the tool. We found nutrition information in only five (3.5%) fast food stores. None of the full-service restaurants had nutrition information available for costumers. Differences across neighborhoods were not as clear, albeit we were able to show a trend for a few components that favored high-income neighborhoods. For instance, restaurants located in high-income neighborhoods were more likely to have salads and fruits on the menu (Table 3).

**Table 3:** ESAO Restaurants Observation Tool measures per store type and neighborhood income.a Sao Paulo, Southeastern Brazil, 2011.

Item content	Store type		Neigborhood household median income		

	Full-service restaurants (n = 114)	Fast-food restaurants (n = 143)	Low-income neighborhoods (n = 181)	Middle-income neighborhoods (n = 210)	High-income neighborhoods (n = 81)

	%	%	%	%	%
Salad availability on menu	89.5	44.1^c^	39.8	57.4	58.0^d^
Salad bar availability	51.8	4.2^ c^	16.0	14.3	19.8
Fresh fruit availability	44.7	11.2^ c^	13.3	15.7	28.4^ e^
Freshly squeezed fruit juices availability	75.4	56.6^d^	53.0	61.4	64.2
Fruit and vegetable signage/promotionᵻ	5.3	6.3	3.9	7.6	4.9
Ultra-processed foods signage/promotion	27.2	19.6	24.3	32.9	23.5
All-you-can-eat buffet only^b^	15.8	1.4^ c^	3.3	3.8	9.9
Nutrition facts availability^b^	0.0	3.5	0.0	1.0	3.7
Substitute fries for salad	22.8	19.6	59.6	48.7	53.6
Encourage smaller servings	46.9	32.2^e^	68.8	66.4	54.8
Fruits are cheaper than/or the same price as sugar-rich desserts	63.2	18.2^ e^	50.0	47.8	42.1
Freshly squeezed juices are cheaper than/or the same price as soda	45.3	42.0	48.4	39.2	28.8
Cheaper combos	32.7	32.2	59.6	66.7	52.4

^a^ For comparison purposes, analysis included only full-service and fast-food restaurants assessed. Other types of restaurant-like stores, such as bars, were excluded.

^b ^Fisher nonparametric test performed due to insufficient count.

^c^ p < 0.001.

^d^ p < 0.01.

^e^ p < 0.05.

Supermarkets, large chain grocery stores, open-air food markets and specialty fruit and vegetable stores, when compared with locally-owned grocery stores and corner stores, had significantly higher availability and variety of fresh produce, but lower availability of ultra-processed foods (i.e., soda and corn chips). Signage or promotion of ultra-processed foods was uniformly found across stores, while fruit and vegetable promotion was 13 times less likely to be found at locally-owned grocery stores and corner stores. When the tool components were compared according to neighborhood median household income (considering the neighborhood where the store was located), we only found similarities regarding fresh produce. However, lower and middle-income neighborhoods had a greater availability of soda, fruit-flavored drink mixes, and chips, but lacked sugar-sweetened nectars or juices, which were more likely to be found in higher income neighborhoods (Table 4).

**Table 4:** ESAO Food Store Observation Tool measures per store type and neighborhood income.a Sao Paulo, Southeastern Brazil, 2011.

Item content	Store type		Neighborhood household median income		

	Supermarkets, large chain grocery stores, open-air food markets and FV specialized stores or markets (n = 24)	Locally-owned grocery stores or corner stores (n = 253)	Low-income neighborhoods (n = 101)	Middle-income neighborhoods (n = 106)	High-income neighborhoods (n = 106)
Fresh produce availability (%)	100.0	22.5^c^	19.5	31.7	25.0
Orange	100.0	15.4^ c^	16.3	22.5	22.9
Banana	91.7	17.4^ c^	17.1	23.9	22.9
Papaya	95.8	13.4^ c^	13.8	20.4	22.9
Apple	95.8	13.4^ c^	13.0	22.5	18.8
Tomato	91.7	17.8^ c^	17.1	25.4	15.3
Onions	91.7	20.6^ c^	19.5	26.8	25.0
Carrot	95.8	15.8^ c^	16.3	23.2	20.8
Lettuce	87.5	13.8^ c^	16.3	19.0	18.8
Fresh fruits and vegetable located near the entrance of the store (%)	79.2	75.4	75.0	82.2	58.3
Fresh produce variety (number) - Mean (SD)					
Fruits	17.4 (6.2)	1.8 (4.6)^ c^	2.0 (5.1)	3.3 (6.4)	3.5 (7.5)
Vegetables	16.8 (4.6)	11.8 (4.7)^ c^	13.5 (4.9)	13.0 (4.8)	18.1 (5.4)
Availability of ultra-processed foods					
Soda	62.5	82.2^e^	82.9	83.1	64.6^e^
Sugar-free soda	62.5	62.8	63.9	58.8	70.2
Sugar-sweetened nectar/juice	25.0	66.4^ c^	27.6	30.3	47.9^e^
Fruit-flavored drink mix	25.0	63.6^ c^	61.8	54.9	39.6^e^
Chocolate sandwich cookies	33.3	68.8^d^	71.5	59.9	60.4
Corn chips	45.8	82.2^ c^	88.6	70.4	68.8^ d^
Signage/promotion (%)					
Fruit and vegetables signage/promotion	62.5	4.7^ c^	4.1	12.0	10.4
Uprocessed foods signage	25.0	24.1	20.5	25.7	34.0
Food prices (R$)^b^ - Mean (SD)					
Fruits (kg)	2.57 (2.34)	2.34 (0.65)	2.14 (0.57)	2.52 (2.22)	2.71 (0.46)
Vegetables (kg)	2.79 (1.15)	2.10 (0.80)^e^	1.96 (0.55)	2.61 (1.21)	2.53 (0.88)
Soda (350 ml)	1.27 (0.41)	1.89 (0.47)^ c^	1.84 (0.51)	1.94 (0.46)	2.20 (0.64)^e^
Sugar free soda (350 ml)	1.38 (0.37)	2.06 (0.37)^ c^	2.04 (0.35)	2.03 (0.46)	2.31 (0.59)^e^
Fruit-flavored drink mix (1 unit)	0.52 (0.17)	0.68 (0.27)	0.70 (0.28)	0.65 (0.27)	0.85 (0.45)^e^
Sugar-sweetened nectar/juice (1 L)	3.02 (0.78)	3.83 (1.02)^d^	3.78 (0.95)	3.88 (1.16)	4.23 (1.48)
Chocolate sandwich cookies (180 g)	0.99 (0.21)	1.35 (0.58)^e^	1.34 (0.60)	1.35 (0.60)	1.65 (0.71)^e^
Corn chips (30 g)	1.30 (0.29)	1.12 (0.04)	1.06 (0.05)	1.18 (0.06)	1.75 (0.14)^ c^

^a^ For comparison purposes, analysis included only supermarkets/fruits and vegetables specialized stores and locally owned grocery stores/corners stores. Other types of retail food stores, such as conveniences stores, were excluded.

^b^ As of Jan 2011, R$1.70 = US$1.00.

^c^ p < 0.001.

^d^ p < 0.01.

^e^ p < 0.05.

Soda and sugar-sweetened nectars or juices were more expensive at locally-owned grocery stores and corner stores. Fruit-flavored drink mixes prices, on the other hand, did not vary across neighborhoods and had the lowest mean prices among all the assessed foods. While fruit and vegetable mean prices were similar across neighborhoods, most of the assessed processed foods were cheaper in lower and middle-income neighborhoods – leading to higher relative prices of fruits and vegetables in lower and middle-income neighborhoods when compared with more affluent neighborhoods in the city (Table 4). We did not find any differences in fresh produce quality across store types or neighborhoods.

## DISCUSSION

The tools presented here (ESAO-R and ESAO-S) were reliable and, despite including fewer items than previous published tools, they were able to discriminate across store types and neighborhoods of different median incomes. In comparison with similar instruments that had previously produced good to high reliability scores,^15,18,27-29^ we obtained similar scores, which suggests limited changes in the measures over several weeks. Our tools can reliably assess the availability, variety, pricing, quality, and promotion of foods within retail food stores, as well as the facilitators and barriers to healthy eating in restaurants in a large urban area of Brazil.

Significant differences in food environment variables across retail food stores and restaurants showed the utility of our tools, as well as in neighborhoods across the income spectrum, which can be interpreted as support for the tools’ construct validity. Observers presented high levels of agreement on most items.

The unique contribution of this study was, therefore, to present reliable measures with sound construct validity, which included items widely used in the literature to assess micro-level food environments adapted to the Brazilian context. These tools were more time and cost-saving than the ones previously reported, considering we were able to reduce the number of questions – still being able to discriminate across different contexts. In addition, we were able to include more relevant foods to the local epidemiological context.^23^ Based on a tool used to collect in-stores measures to evaluate the environmental determinants of cardiovascular diseases in five different countries,^6^ we decided to include in our tool measures of in-store signage and promotion.

However, food signage and promotion had either low reliability or the Kappa coefficients could not be estimated because of the limited availability of signage in stores, which was also true for items that evaluated facilitators of healthier choices. These findings indicate the difficulties that consumers face in selecting healthy foods in restaurants in Sao Paulo. For instance, only five fast-food restaurants and no buffet style or full-service restaurants had readily available nutrition information, which is even lower than what had been previously reported in the United States.^29^ The implementation of recent measures to increase the availability of nutrition information in other cities^10,26^ may influence local policy making to improve nutrition information availability in Sao Paulo. Future changes in the Brazilian food environment deserve further analysis and ESAO tools could contribute with that.

Previous findings regarding in-store measures in Brazil are restricted to retail food stores.^22^ However, eating out in Brazil already accounts for 17.0% and 29.0% of all calories consumed by low and high-income Brazilians, respectively.^3^ The more Brazilians increase their frequency of away-from-home eating, especially those living in large cities, the more exposed they will be to food environments that may encourage them to choose unhealthy foods. Few interventions in restaurants have found that increasing the number of healthier choices^21^ and providing nutrition information^10^ help shift purchase intentions away from unhealthier options.

Our findings for retail food stores agree with findings from another Brazilian city,^22^ which indicate that more ultra-processed foods are found in lower-income neighborhoods. Fruits and vegetables, however, were found in four times as many supermarkets or fruit and vegetable markets than in locally-owned grocery stores or corner stores, which were also more likely to be found in low-income neighborhoods.^11^ Differences in in-store environments may affect food purchasing and health-related outcomes, and contribute to disparities in diet-related diseases.^8^ In fact, we found similar availability, quality and price of fruits and vegetables across the city. Nonetheless, ultra-processed foods were more likely to be found in lower-income neighborhoods, and most of the products assessed were cheaper in the most deprived areas of the city.

Sugar-sweetened beverages were cheaper in lower-income neighborhoods, except for sugary processed juices. It is possible that high-income individuals may be choosing more expensive sugar-sweetened beverages, such as processed juices and nectars, rather than soda and cheaper fruit-flavored drinks. Social marketing aimed at informing consumers of the problems associated with all types of sugar-sweetened beverages – and not only soda – could be considered by local governments.

The limitations of this study include the restriction to 13 districts within a single metropolitan region in Brazil. Though the present data should not be considered representative of the city or the country, the selected neighborhoods provided a representative variation of the city’s socioeconomic status. The median monthly household income in each census tract varied from R$1,020 to R$9,500.^d^ Sampled census tracts sizes were similar to the city mean number of residents per census tracts.^b^

Applying our measures at other locations would allow for their generalizability of the measures, however new assessments of the psychometric properties of these tools are encouraged if they are adapted to local needs. A limitation of all in-store environment studies is the cost of personnel time, nonetheless, we included fewer items in our instrument compared with previous tools^15,29^ and yet they discriminated across store types and neighborhoods in similar ways.

Given the important public health task to fight increasing rates of obesity in Brazil,^25^ and the difficulty in maintaining a healthy weight in unsupportive environments,^4^ high-quality and context specific micro-level food environment observation tools are required. The measures of the food environment presented here are feasible, reliable, and discriminated across store types and neighborhoods of different incomes. They are applicable in a variety of similar urban settings to test associations of urban food environments and food intake, when studying social determinants of health, in intervention studies, and in evaluations of changing urban food environments. Our tools also provide a method for future evaluative research of environmental policy interventions in low and middle-income countries.

## Footnotes

a Instituto Brasileiro de Geografia e Estatística. Pesquisa de Orçamentos Familiares 2008-2009: despesas, rendimentos e condições de vida. Brasília (DF); 2010 [cited 2015 Apr 18]. Available from: http://www.ibge.gov.br/home/estatistica/populacao/condicaodevida/pof/2008_2009/POFpublicacao.pdf

b ESAO Restaurants Observation Tool (ESAO-R) and ESAO Food Store Observation Tool (ESAO-S) and manuals can be requested from the first author by e-mail.

c Instituto Brasileiro de Geografia e Estatística. Censo 2010. Brasília (DF); 2011 [cited 2015 Apr 18]. Available from: http://www.ibge.gov.br/home/estatistica/populacao/censo2010/default_resultados_universo.shtm

d Considering Jan 2011, R$1.70 = US$1.00.
